# Adenovirus Pneumonia Complicated With Acute Respiratory Distress Syndrome: A Case Report

**DOI:** 10.1097/MD.0000000000000776

**Published:** 2015-05-22

**Authors:** Ka-Ho Hung, Lung-Huang Lin

**Affiliations:** From the Department of Pediatrics (K-HH, L-HL), Cathay General Hospital, Taipei; and School of Medicine (L-HL), Fu-Jen Catholic University, New Taipei City, Taiwan.

## Abstract

Severe adenovirus infection in children can manifest with acute respiratory distress syndrome (ARDS) and respiratory failure, leading to the need for prolonged mechanical support in the form of either mechanical ventilation or extracorporeal life support. Early extracorporeal membrane oxygenation (ECMO) intervention for children with ARDS should be considered if selection criteria fulfill.

We report on a 9-month-old boy who had adenovirus pneumonia with rapid progression to ARDS. Real-time polymerase chain reaction tests of sputum and pleural effusion samples confirmed adenovirus serotype 7. Chest x-rays showed progressively increasing infiltrations and pleural effusions in both lung fields within 11 days. Because conventional ARDS therapies failed, we initiated ECMO with high-frequency oscillatory ventilation (HFOV) for 9 days. Chest x-rays showed gradual improvements in lung expansion.

This patient was subsequently discharged after a hospital stay of 38 days. Post-ECMO and adenovirus sequelae were followed in our outpatient department.

Adenovirus pneumonia in children can manifest with severe pulmonary morbidity and respiratory failure. The unique lung recruitment by HFOV can be a useful therapeutic option for severe ARDS patients when combined with sufficient lung rest provided by ECMO.

## INTRODUCTION

Adenovirus infections occur primarily in infants and children <5 years of age and account for 2% to 5% of respiratory illnesses among pediatric patients and 4% to 10% of childhood pneumonia cases. Although most children with an adenovirus infection develop mild upper respiratory tract disease, more severe cases may occur with lower respiratory tract involvement. Here, we report a case of a 9-month-old boy who presented with progressive respiratory failure because of a severe lower respiratory tract adenovirus infection. He was treated by extracorporeal membrane oxygenation (ECMO) and high-frequency oscillatory ventilation (HFOV) for 9 days. He was subsequently discharged without oxygen therapy at 38 days after his admission.

## METHOD

This was a case report. The Institutional Review Board of the Cathay General Hospital, Taipei, Taiwan, approved this study. Informed consent was obtained from the patient.

## CASE REPORT

**FIGURE 1:**
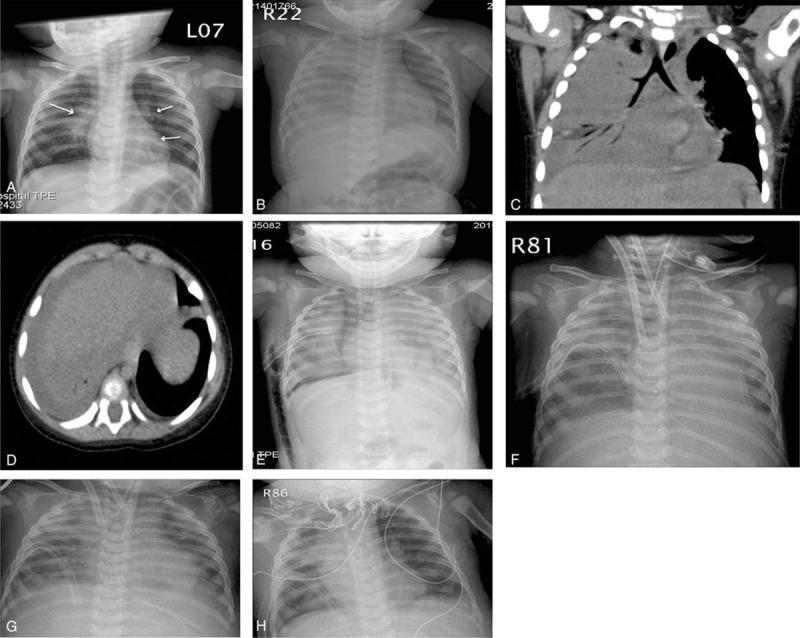
Changes in lung conditions observed on chest x-rays and computed tomography (CT) scans. (A) Chest x-ray on hospital day 1 showed increased bilateral perihilar infiltrations. (B) Chest x-ray on hospital day 5 showed increased infiltration in the left lung field and whiteout in the right lung field with effusion. (C) CT images on hospital day 5 revealed diffuse infiltration in the right lung field with partial collapse and pleural effusion. (D) Sagittal view of chest CT image showing the same findings as (C). (E) Chest x-ray on hospital day 11 showed a chest tube inserted over the right side and progressively increasing infiltrations in bilateral lung fields. (F) Chest x-ray on hospital day 11 after introducing venoarterial ECMO combined with HFOV. (G) Chest x-ray on hospital day 17 after introducing venovenous ECMO revealed improvements in bilateral lung expansion while under EMCO and HFOV. (H) Chest x-ray on hospital day 20 after removing ECMO showed better lung recruitment with reduced bilateral infiltrations. ECMO = extracorporeal membrane oxygenation, HFOV = high-frequency oscillatory ventilation.

A 9-month-old baby boy who had previously been hospitalized because of acute bronchiolitis was admitted to our Department of Pediatric for recurrent respiratory distress management. His initial white blood cell count was 14.98 × 10^3^ cells/mm^3^ with 73.4% segments and a C-reactive protein (CRP) level of 7.904 mg/dL. Supportive care with intravenous cefuroxime was initially given. On the sixth day in a general hospital ward, his breathing worsened associated with subcostal retraction and frequent desaturation. A chest x-ray, echogram, and a computed tomography (CT) scan revealed consolidation with pleural effusion in his right lung and significant infiltrations in the left lung field (Fig. 1A–D). Thus, he was transferred to the Pediatric Intensive Care Unit (PICU) for thoracocentesis and intensive care management. Prior to the transfer, he tested positive for adenovirus infection by throat swab viral culture and a nasal smear adenovirus antigen test, but was negative for respiratory syncytial virus and influenza virus antigen tests.

Initial therapy for progressive respiratory failure included minimal oxygen administration, inhaled β-adrenergic agonists, intensive chest care, and antibiotics. Shortly after his PICU admission, he displayed a consciousness disturbance and we performed a spinal tap. Viral and bacterial cultures of cerebral spinal fluid did not show any growth for pathogens. Additionally, sputum, pleural effusion, and serum bacterial cultures were negative. A urine pneumococcus antigen test was also negative.

On hospital day 11 (PICU day 6), he presented with low-grade fever, air hunger, and frequent cyanosis that required intubation with controlled mandatory ventilation (CMV). Arterial blood gas analysis showed carbon dioxide retention and a reduced ratio of partial pressure of oxygen in arterial blood (Pao_2_: 68) to the fraction of inspired oxygen (Fio_2_: 0.6), Pao_2_/Fio_2_ of <200. Along with worsened bilateral infiltrates on a chest x-ray (Fig. 1E), acute respiratory distress syndrome (ARDS) was diagnosed and we changed the ventilation mode from CMV to high-frequency oscillatory ventilation (HFOV) with a mean airway pressure (MAP) of 20 cmH_2_O, an amplitude of 32 cmH_2_O, and a frequency of 8 Hz. However, poor serum arterial oxygen saturation (Sao_2_: 70%–80%) and hemodynamics remained despite this support.

We then introduced a venoarterial (V-A) mode of ECMO for oxygen support and cardiopulmonary rest (Fig. 1F). HFOV with MAP of 18 cmH_2_O, amplitude of 32 cmH_2_O, and frequency of 8 Hz was maintained during ECMO. In addition to mechanical support, we tried several treatments for ARDS, including positional changes for better lung recruitment and drugs, including antimicrobial agents (vancomycin, ceftazidime, and oseltamivir), Primacor (Sanofi-Aventis, Paris, France) for pulmonary hypertension, and dopamine for better hemodynamics.

On hospital day 17 (PICU day 12), V-A ECMO was changed to venovenous (V-V) ECMO because of improved hemodynamics and lung expansion seen on a chest x-ray (Fig. 1G). However, his course was further complicated by acute renal insufficiency, which recovered spontaneously without using peritoneal dialysis. Additionally, sputum and pulmonary exudate samples were analyzed by real-time polymerase chain reaction, which revealed adenovirus serotype 7 (sputum: 5.3 × 10^8^ copies/mL; pleural effusion: 4.8 × 10^7^ copies/mL). The patient was successfully decannulated from ECMO on hospital day 20 (PICU day 15) after a 9-day course (Fig. 1H).

Improved pulmonary oxygenation was confirmed with a Pao_2_/Fio_2_ ratio of 442.4 determined after V-V ECMO was discontinued. We then changed from HFOV to CMV (rate: 25; peak inspiratory pressure: 22; positive end expiratory pressure: 4; and Fio_2_: 0.25) and continued the weaning process. His feeding began smoothly after removal from ECMO. Mechanical ventilation was discontinued on hospital day 30 (PICU day 25). He was moved to a general ward on hospital day 32 and was subsequently discharged 6 days later without oxygen therapy.

## DISCUSSION

Adenovirus has been implicated as the etiologic pathogen in 5% to 11% of bronchitis cases, 2% to 10% of bronchiolitis cases, and 4% to 10% of pneumonia cases in infants and children.^1,2^ Serotypes 3, 7, 7a, and 21 are the most common causes of lower respiratory disease and are associated with severe disease as well as serious pulmonary sequelae such as bronchiectasis, bronchiolitis obliterans, unilateral hyperlucent lung, and persistent abnormal pulmonary function.^3–5^ Severe adenovirus infection mimics bacterial infection in its clinical, laboratory, and radiographic features.^6^ Our patient had adenovirus serotype 7 infection with severe multilobar pneumonia and pleural effusions. Initial leukocytosis and an elevated CRP level made it difficult to distinguish this from a bacterial infection.

It has been postulated that mechanical assistance may cause lung injury and increase the risk of postinfectious bronchiolitis obliterans.^7^ Our initial treatment goal was to establish adequate oxygenation without using ventilator support, including a nasal cannula or an oxygen mask. Intensive chest percussion and a second-line antibiotic (cefuroxime) were administered to treat common bacterial LRIs (lower respiratory tract infections). Because ARDS had developed and progressed, we introduced HFOV as a theoretically better type of ventilation as compared with CMV. The potential advantages of HFOV over CMV include delivering a smaller tidal volume (1–3 mL/kg), thus limiting alveolar overdistension; applying a higher MAP than that with CMV, thus promoting more alveolar recruitment; and maintaining a constant MAP during inspiration and expiration, thus preventing end-expiratory alveolar collapse.^8^

We believe that our patient had pulmonary function recovery within the short period of 9 days primarily because of the unique lung recruitment capability of HFOV, which was used in combination with ECMO. This was reflected by the subsequent smooth weaning from HFOV ventilation to CMV with low settings.

ECMO has been used as rescue therapy for >2 decades for children with ARDS and with reported survival rates of >50%.^9^ Peek et al^10^ suggested that the primary benefit of ECMO was resting the lungs from high pressure and Fio_2_ ventilation, thus minimizing iatrogenic contributions to lung injury. Based on Children's Health Care of Atlanta ECMO selection criteria, we initiated ECMO for our patient due to an acute, life-threatening but potentially reversible event and the failure of conventional ARDS therapies. V-A ECMO was applied because of his unstable hemodynamics at the early stage of ARDS and was then changed to V-V ECMO 6 days later. Common complications associated with ECMO, such as bleeding and technical errors, were avoided because of the short duration with this setup. However, our patient presented with acute renal insufficiency 2 days after beginning ECMO, which recovered spontaneously without using peritoneal dialysis.

Regarding neurological deficits after ECMO, brain sonography showed normal brain development without any previous hemorrhage tracts, and his mental and motor development is being observed at our outpatient department. We recommend that early intervention with ECMO for the patient successfully preserved his pulmonary function from progressing to ARDS by providing time to rest his lungs, thus protecting and efficiently recovering lung function with the aid of HFOV.

In conclusion, adenovirus pneumonia in children can manifest with severe pulmonary morbidity and life-threatening respiratory failure, which results in the need for prolonged mechanical support either by mechanical ventilation or extracorporeal life support. Early ECMO intervention for children with ARDS should be considered if the selection criteria are fulfilled. Although HFOV has not shown any benefits in terms of ARDS patient mortality,^11–13^ the unique lung recruitment by HFOV can be a useful therapeutic option for severe ARDS patients when combined with sufficient lung rest provided by ECMO. Owing to the possibility of developing pulmonary sequelae, long-term follow-up is suggested.
